# Emergent salvage surgery for massive hemoptysis after proton beam therapy for lung cancer: a case report

**DOI:** 10.1186/s40792-021-01177-9

**Published:** 2021-04-20

**Authors:** Haruaki Hino, Kahori Nakahama, Makoto Ogata, Kayoko Kibata, Chika Miyasaka, Takahiro Utsumi, Natsumi Maru, Hiroshi Matsui, Yohei Taniguchi, Tomohito Saito, Koji Tsuta, Tomohiro Murakawa

**Affiliations:** 1 Department of Thoracic Surgery, Kansai Medical University, 2-3-1 Shinmachi Hirakata-shi, Osaka, 573-1191 Japan; 2 First Department of Internal Medicine, Division of Thoracic Oncology, Kansai Medical University, 2-3-1 Shinmachi Hirakata-shi, Osaka, 573-1191 Japan; 3 First Department of Internal Medicine, Division of Respiratory Medicine, Infectious Disease and Allergology, Kansai Medical University, 2-3-1 Shinmachi Hirakata-shi, Osaka, 573-1191 Japan; 4 Department of Pathology and Laboratory Medicine, Kansai Medical University, 2-3-1 Shinmachi Hirakata-shi, Osaka, 573-1191 Japan

**Keywords:** Salvage surgery, Massive hemoptysis, Broncho-pulmonary artery fistula, Proton beam therapy

## Abstract

**Background:**

Salvage surgery is an effective therapeutic option for patients experiencing relapses after chemoradiotherapy for advanced-stage lung cancer or after high-dose radiotherapy for early-stage lung cancer. We report a case involving an emergent salvage surgery for a patient with massive hemoptysis who developed lung cancer recurrence after undergoing proton beam therapy 7 years prior to presentation.

**Case presentation:**

A 70-year-old male patient was emergently admitted due to massive hemoptysis. He had undergone proton beam therapy for a stage I adenocarcinoma of the left upper lobe 7 years ago, and was receiving chemotherapy for local recurrence. We performed an emergent salvage pulmonary resection to achieve hemostasis. During the operation, we confirmed the presence of a left broncho-pulmonary arterial fistula, which was considered as the origin of the massive hemoptysis. We repaired the fistula between the pulmonary artery and left upper bronchus without incident; an orifice of the fistula at the left pulmonary artery was sutured using a non-absorbable monofilament, and the central portion of the orifice of the fistula at the left upper bronchus was closed with a mechanical stapling device. The postoperative diagnosis was of an adenocarcinoma—ypT3(pm1) N0M1a (dissemination)-IVA, ef1b. The patient has survived for over a year with the cancer in almost complete remission following the administration of an epidermal growth factor receptor tyrosine kinase inhibitor.

**Conclusions:**

Emergent salvage surgery demands high skill levels with optimal timing and correct patient selection. Our case suggested that the procedure played an important role in controlling serious bleeding and/or infectious conditions. Consequently, he could receive chemotherapy again and survive for over a year.

**Supplementary Information:**

The online version contains supplementary material available at 10.1186/s40792-021-01177-9.

## Background

Salvage surgery is an effective therapeutic option for patients with relapses after chemoradiotherapy for advanced-stage lung cancer or after high-dose radiotherapy for early-stage lung cancer, especially under inoperable conditions [1–7]. Complications of these therapies, such as massive hemoptysis, lung abscess, or empyema, require timely intervention. We report a case of an emergent salvage surgery for a patient with massive hemoptysis who had lung cancer recurrence after undergoing proton beam therapy (PBT) 7 years before recurrence.

## Case presentation

**Fig. 1:**
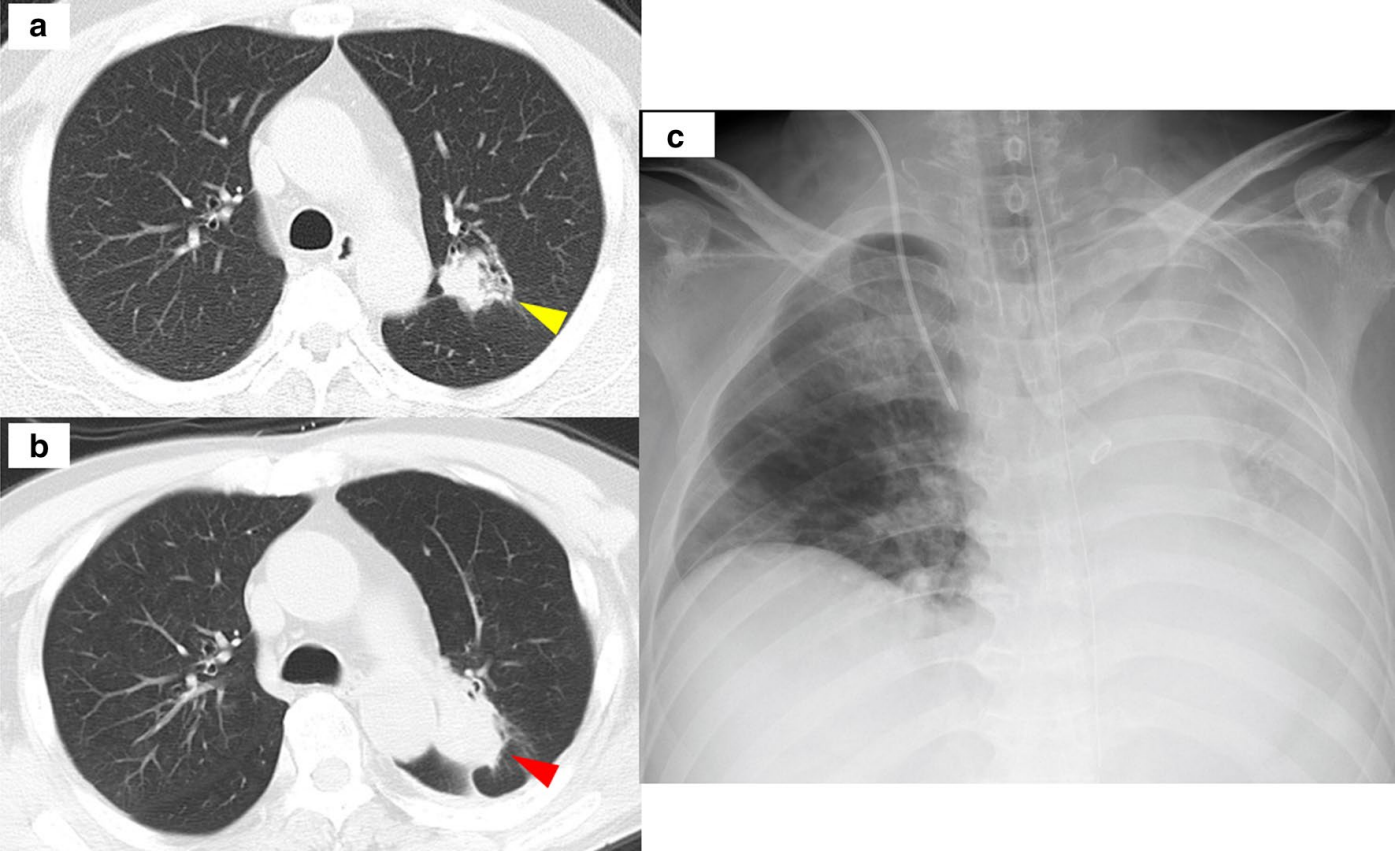
Preoperative imaging. **a** Computed tomography before proton bean therapy (yellow arrowhead). **b** Computed tomography of lung cancer recurrence (red arrowhead). **c** Chest X-ray after massive hemoptysis. *PBT* proton beam therapy

**Fig. 2:**
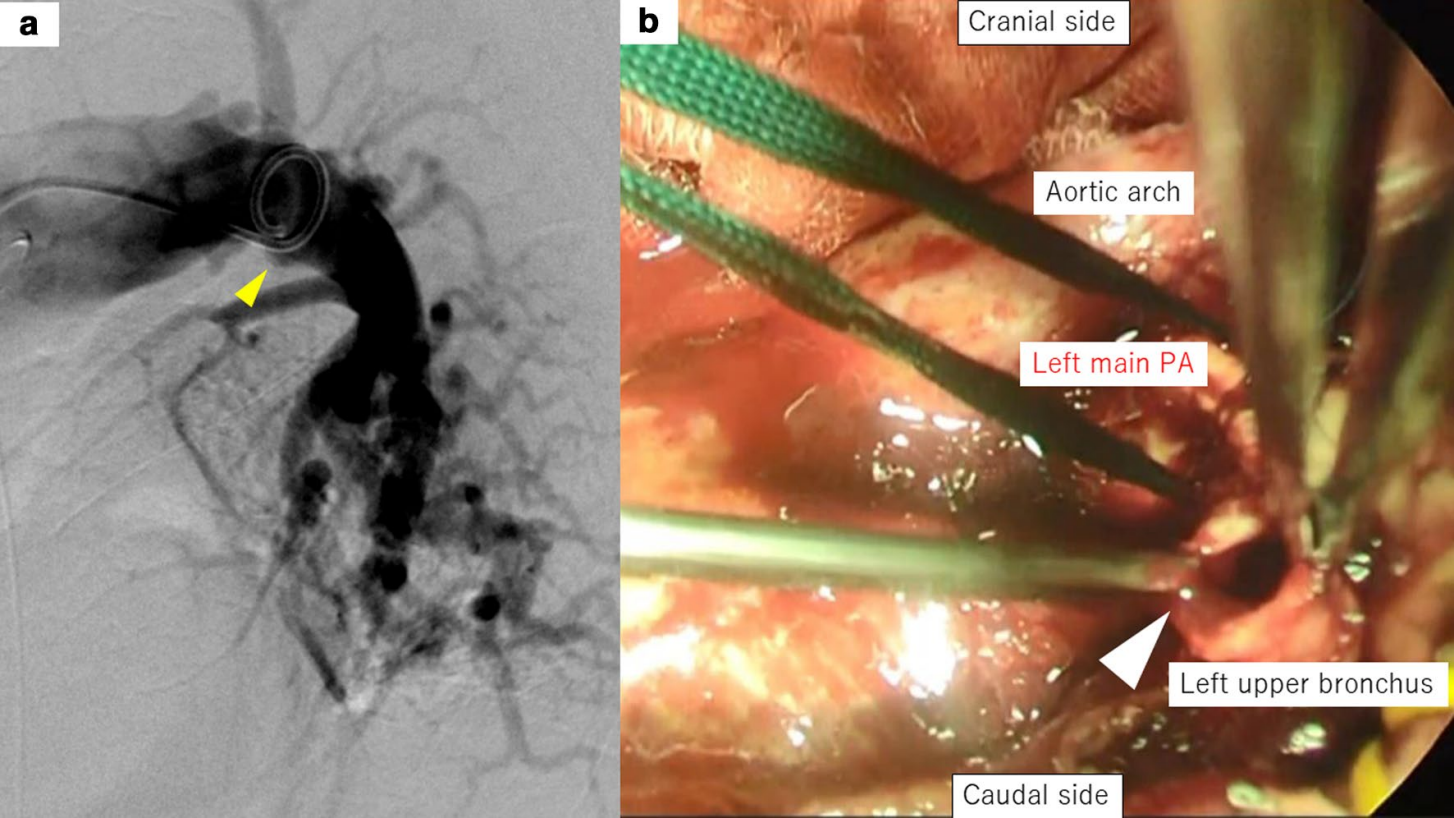
Preoperative and perioperative imaging. **a** Left pulmonary artery angiography reveals a pulmonary artery aneurysmal change (yellow arrowhead). **b** Intraoperative view of the bleeding from the pulmonary artery through the BPAF (white arrowhead). *BPAF* broncho-pulmonary artery fistula, *PA* pulmonary artery

**Fig. 3:**
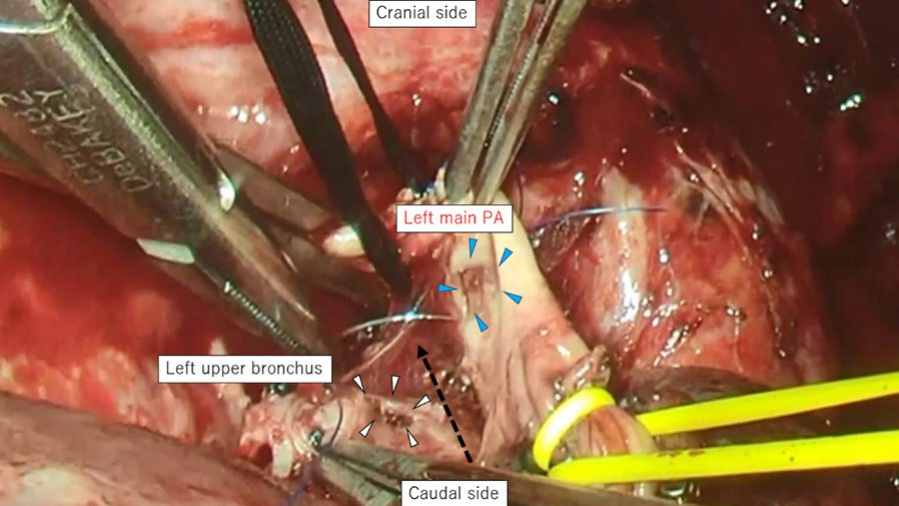
Intraoperative imaging. Intraoperative view of BPAF repair on the pulmonary artery side, which involved the manual closure of the fistula with an unabsorbable monofilament material. Blue arrowheads show the BPAF from the pulmonary artery side, and white arrowheads indicate the BPAF from the bronchus side. The dotted line suggests the line along which cutting was performed with an endostapler in the left upper bronchus to repair the BPAF from the bronchus side. *BPAF *broncho-pulmonary artery fistula, *PA* pulmonary artery

**Fig. 4:**
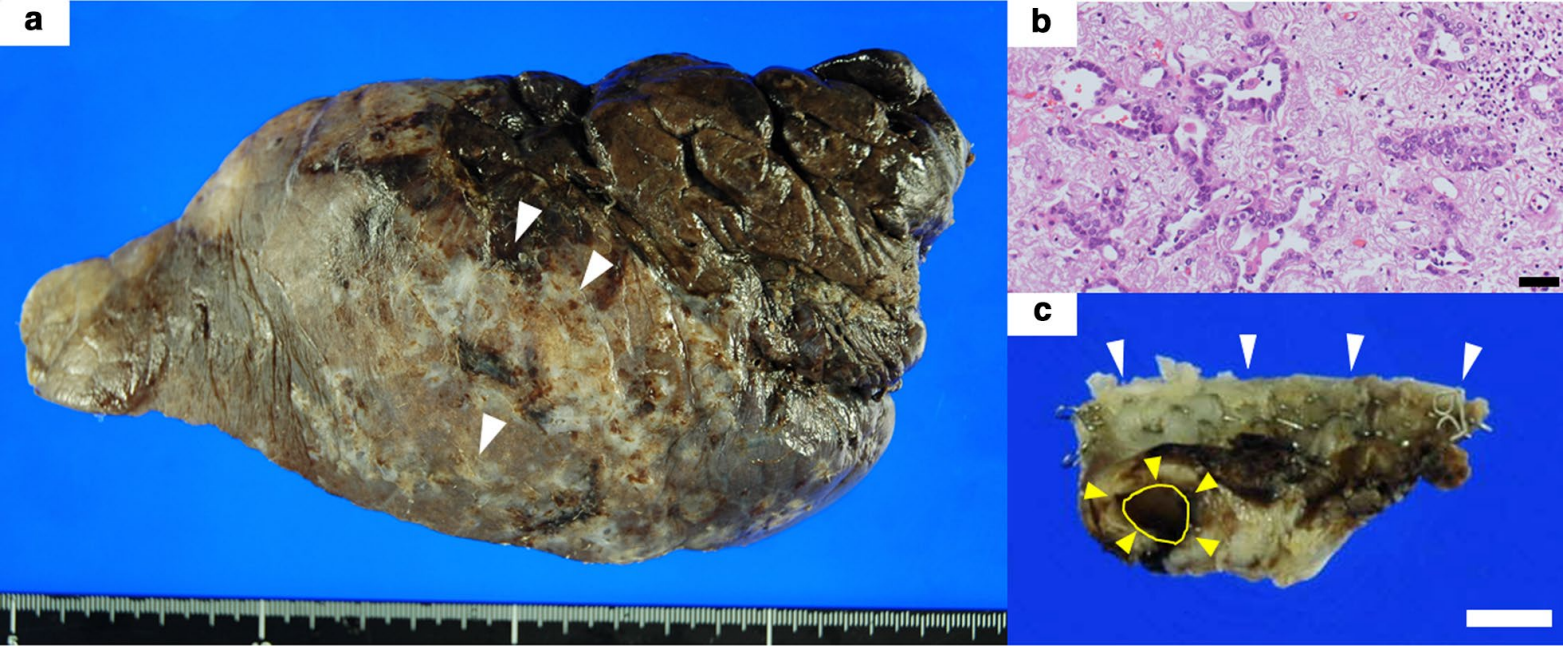
Macroscopic and microscopic views of the resected lung. **a** Macroscopic view of the resected lung with dissemination (white arrowheads). **b** Hematoxylin and eosin staining of the adenocarcinoma, which included fibrotic change due to prior treatment with PBT and chemotherapy. Bar = 50 µm. **c** Macroscopic view of the resected bronchus with fistula (yellow arrowheads and circle). White arrowheads show the central side of the left upper bronchus with staples. Bar = 5 mm. *PBT* proton beam therapy

A 70-year-old man receiving chemotherapy for lung cancer recurrence was admitted to our emergency department due to massive hemoptysis. Seven years prior to this presentation, he underwent PBT (72.6 Gy in 22 fractions) for a stage I adenocarcinoma located peripherally in the left upper lobe, because he had refused radical surgery (Fig. 1a). When local recurrence was diagnosed after 4 years (Fig. 1b), chemotherapy with erlotinib and bevacizumab was administered for approximately 2 years, during which his condition was stable. Shortly prior to admission, the patient had experienced a few minor episodes of hemoptysis, and bevacizumab was discontinued. Three months later, he was admitted to our intensive care unit with massive hemoptysis. He underwent left-sided intubation with a double-lumen tube to protect the contralateral lung from the massive bleeding from the left upper lobe (Fig. 1c). Bronchial artery embolization failed to detect a bleeding source, but an aneurysmal change in the pulmonary artery was found, which was thought to be the possible culprit (Fig. 2a). Although the bleeding had stopped momentarily, it could have resumed at any time. Therefore, we decided to perform an emergent salvage surgery. Intraoperatively, after left upper lobectomy with pulmonary arterioplasty was performed, we noticed active bleeding from the open orifice of the left upper bronchus (Fig. 2b and Additional file 1: Video S1). Close observation revealed that the bleeding originated from the left main pulmonary artery through a broncho-pulmonary arterial fistula (BPAF). We closed the fistula on the pulmonary artery side with a non-absorbable monofilament suture (Fig. 3). We also stapled off the left upper bronchus at the central side of the fistula using a mechanical stapling device (Covidien Endo GIA™ Tri Staple™ 2.0 Black Cartridge 45 mm, Medtronic, Fridley MN) (Fig. 3 dotted line). The operative time was 179 min and the estimated blood loss was 496 mL. The patient received an intraoperative blood transfusion (red blood cells: 560 mL, fresh frozen plasma: 480 mL). The postoperative diagnosis was of an adenocarcinoma—ypT3(pm1) N0M1a (dissemination)-IVA, ef1b (Fig. 4a, b). Residual cancer was not detected in the left upper bronchus at the BPAF site (Fig. 4c). Postoperatively, the patient developed drug-induced subacute interstitial pneumonia, requiring treatment with oral methylprednisolone. He was discharged on a postoperative day 31 in a stable condition. Therapy with an epidermal growth factor receptor tyrosine kinase inhibitor (osimertinib) was administered for over a year after the surgery, and his condition has remained stable with the tumor showing almost complete remission.

## Discussion

This is the first report of an emergent salvage surgery for lung cancer recurrence in the setting of BPAF development after PBT. Recently, the need for salvage procedures after definitive chemotherapy and/or radiotherapy for early or advanced lung cancer has been increasing, partly because there are more patients undergoing chemotherapy as a primary treatment and partly because newer effective chemotherapeutic drugs, such as immune checkpoint inhibitors, have become available. Furthermore, the number of patients undergoing high-dose radiation therapies such as stereotactic radiotherapy (SBRT), carbon ion therapy (CIT), and PBT, has also been increasing [6, 7]. For high-energy therapy, including PBT, SBRT, and CIT, an acceptable outcome was reported with a 3-year local control rate of 95.7% for stage I lung cancer [8, 9]. However, the long-term feasibility is unknown, because the inoperable patients did not survive in the long term due to poor general health conditions. One study reported that a patient with peripherally located lung cancer who had undergone SBRT developed massive hemoptysis after 4 years and was successfully treated with bronchial artery embolization [10]. Therefore, late-onset adverse events such as massive hemoptysis might be possible, such as in the present case.

Salvage surgery is generally a demanding and challenging procedure that requires experienced postoperative patient care; however, clinical experience is scarce, because the indication for salvage surgery is limited to selected patients, so as to perform a complete resection safely. There is no consensus on the indications for salvage surgery. As for the definition of salvage surgery, prior reports have described three categories [1, 11]: (I) salvage surgery for local recurrence after SBRT for early-staged lung cancer; (II) salvage procedure for local recurrence or a persistent tumor after full-dose chemoradiotherapy for locally advanced lung cancer, and (III) emergent lung resection for serious adverse events of hemoptysis, an uncontrollable lung abscess, or empyema during/after chemotherapy and/or radiotherapy for lung cancer. Based on recently published data, surgical outcomes of salvage surgeries from categories (I) and (II) have been reported; the rates of postoperative morbidity, mortality, and 5-year overall survival were 18.9–25%, 0–4.8%, and 79.5% for category (I), respectively, and 7.9–40.0%, 0–6.7%, and 40.6–53.3% for category (II), respectively [2–9]. Although the short- and long-term results of elective salvage surgeries from categories (I) and (II) were considered feasible and acceptable, the outcomes of salvage surgeries from category (III) are less clear. The results of three emergent salvage surgeries in terms of morbidity, mortality, and mean survival duration were 100%, 0%, and 13.5 ± 5 months, respectively, which were considered to be comparable to those of other types of salvage surgeries (categories [I] and [II]) [10]. In the present case, although the first aim of lung resection was palliation, the patient was able to receive chemotherapy again following the emergency lifesaving surgery and achieved survival for over a year. Therefore, we suggest that emergent salvage lung resection played an important role in controlling the serious life-threatening event of massive hemoptysis and saved the patient’s life, consequently enabling him to receive chemotherapy again.

BPAF is a rare critical event caused by various etiologies including infection, postoperative complications of pulmonary resection and transplantation, and chemotherapy [12, 13]. The clinical manifestation of BPAF is massive hemoptysis, and a delayed response may lead to fatal outcomes [12]. As for the etiology of the BPAF in the present case, both the hilar pulmonary artery and the left main bronchus were included in the irradiation field, and lung cancer recurrence was not confirmed at the left upper bronchus. Therefore, we considered that the BPAF may have arisen as an adverse effect of bevacizumab therapy and may have also been a late-onset adverse effect of high-energy therapy (PBT), which may have affected the event indirectly. The patient fortunately survived due to timely surgical intervention. The diagnosis of BPAF prior to surgery is very challenging, and in clinical practice, many patients with massive hemoptysis of unknown origin can die due to undiagnosed BPAF. Emergent salvage surgery should always be considered for massive hemoptysis due to a possible BPAF after high-energy therapy and/or chemotherapy.

## Conclusions

We performed an emergent salvage lung resection due to massive hemoptysis after PBT for early-staged lung cancer and chemotherapy for local recurrence. Salvage surgery demands high skill levels in addition to optimal timing and patient selection; our results suggest that the procedure played an important role in controlling the life-threatening serious bleeding and/or infectious conditions, which saved the patient’s life. As a result, the patient could receive chemotherapy again, and achieved survival. To elucidate surgical feasibility and long-term survival, a larger series of emergent salvage surgeries for patients with pretreated lung cancer and life-threatening complications should be analyzed in future studies.

## Supplementary Information

**Additional file 1: Video S1.** Bleeding from the broncho-pulmonary arterial fistula (BPAF) in the intraoperative view.

## Data Availability

The datasets supporting the conclusions of this article are available on reasonable request.

## Abbreviations

PBT: Proton beam therapy
BPAF: Broncho-pulmonary artery fistula
SBRT: Stereotactic radiotherapy
CIT: Carbon ion therapy
